# Peer Review of “A Framework for a Statistical Characterization of Epidemic Cycles: COVID-19 Case Study”

**DOI:** 10.2196/27536

**Published:** 2021-03-18

**Authors:** Shelley N Facente

**Affiliations:** 1 United States

**Keywords:** COVID-19, pandemics, infection control, models, experimental, longitudinal studies, statistical modeling, epidemiology

*This is a peer review submitted for the paper “A Framework for a Statistical Characterization of Epidemic Cycles: COVID-19 Case Study.”*

## Round 1 Review

### General Comments

This paper [1] attempts to make some interesting comparisons in epidemic curves but is far too long, requires a thorough copy edit by a native English speaker, and does not appear to be very scientific in nature—it would require a substantial overhaul in both methods and write-up to be suitable for publication in this journal.

### Specific Comments

#### Major Comments

1. I think the paper would benefit from focusing on the central point, with only a minor description of any methods necessary to make that point. For example, most of section 2 (“On the Nature of the Observed Data”) includes a lengthy description of the use of moving averages and R^2^ values, which readers can be expected to understand (and those who do not could learn via appropriate citations). Certainly, the formula for R^2^ is not necessary to include. These types of revisions would make the paper a more readable length.
2. The authors claim the Chinese epidemic cycle has a Gaussian shape, but even in their own drawing it appears much more like a gamma distribution (as would be expected) than Gaussian. They may want to reconsider this section.
3. The crux of this paper seems to be looking for epidemic curves that appear similarly shaped in various countries, and using that to predict the curves for countries where the cycle has not yet reached what the authors term the “MLCE end.” I have never heard of epidemic predictions using a method such as this, and it seems there are many far superior methods for estimating trajectory, so this is a curious choice.
4. Citations require appropriate formatting for journal publications.
